# Isolation and evaluation of Qatari soil rhizobacteria for antagonistic potential against phytopathogens and growth promotion in tomato plants

**DOI:** 10.1038/s41598-023-49304-w

**Published:** 2023-12-12

**Authors:** Amina BiBi, Shazia Bibi, Mohammad A. Al-Ghouti, Mohammed H. Abu-Dieyeh

**Affiliations:** 1 Department of Biological and Environmental Sciences, College of Arts and Sciences, Qatar University, P.O. Box: 2713, Doha, Qatar; 2 Environmental Science Program, Department of Biological and Environmental Sciences, College of Arts and Scieances, Qatar University, P.O. Box: 2713, Doha, Qatar; 3 Biological Science Program, Department of Biological and Environmental Sciences, College of Arts and Sciences, Qatar University, P.O. Box: 2713, Doha, Qatar

**Keywords:** Plant physiology, Environmental sciences, Environmental microbiology, Soil microbiology

## Abstract

Plant growth promoting rhizobacteria are a diverse group of microorganisms that enhance the growth of plants under various conditions. In this study, 55 isolates of endogenous rhizobacteria were collected from the rhizosphere of *Avicennia marina, Suaeda vermiculata, Salsola soda, Anabasis setifera, Salicornia europaea, Arthrocnemum macrostachyum, Limonium axillare, Tetraena qatarensis, Aeluropus lagopoides, and Prosopis juliflora*. The isolates were evaluated in-vitro for their antagonist potential against *Fusarium oxysporum* and *Botrytis cinerea* using the dual culture technique, where the maximum growth inhibition reached 49% and 57%, respectively. In-vivo evaluation was accomplished to determine the growth-promoting potential of the rhizobacteria under greenhouse conditions where the strain ANABR3 (*Bacillus subtilis*) showed the strongest growth-promoting effects. Further in-vivo testing regarding the effectiveness of rhizobacteria in the presence of the phytopathogen was also completed using the Hoagland medium. LEMR3 and SALIR5 (both identified as two strains of *B. subtilis*) supported the tomato seedlings to overcome the disease and significantly (*p* ≤ 0.05) increased above and belowground biomass compared to the control. Additionally, several characterizing tests were carried out on the selected strains, these strains were found to possess numerous features that promote plant growth directly and indirectly such as the production of IAA, HCN, hydrolytic enzymes, ACC deaminase, NH_3,_ and some rhizobacteria were capable of phosphate solubilization. In conclusion, this study showed that local rhizobacterial isolates collected from arid lands possess valuable traits, making them promising bio-control agents and bio-fertilizers for agricultural purposes.

## Introduction

The rhizosphere is the portion of soil that is directly influenced by the plant roots, encompassing root surfaces as well as the strongly adhering soil particles. In this part of the soil system, numerous crucial plant–microbe interactions occur^1^. Various groups of microorganisms inhabit the rhizosphere, with rhizobacteria being the predominant group. These competent microorganisms vigorously colonize all ecological niches within the plant root area^2,3^. Many healthy plants host large numbers of symbiotic and non-symbiotic rhizo-epiphytic and/or endophytic microorganisms. It’s estimated that around 2–5% of rhizobacteria exhibit plant growth-promoting (PGP) characteristics when introduced into a host containing other competing bacterial strains^2^. Consequently, studies are now considering plants to be meta-organisms that harbor close relationships with their associated microorganisms^4^. Research has identified several genera of bacteria as plant growth-promoting rhizobacteria (PGPR), including symbiotic species like *Rhizobium* and non-symbiotic species such as *Bacillus, Pseudomonas*, and *Azotobacter*. Rhizobacteria belonging to these genera are studied worldwide and are currently utilized as bio-inoculants to enhance plant growth and development under different stresses like heavy metal contamination^5^, insecticides^6^, pesticides^7^, hydrocarbon contamination^8^, salinity^9^, and drought^10^.

**Figure 1:**
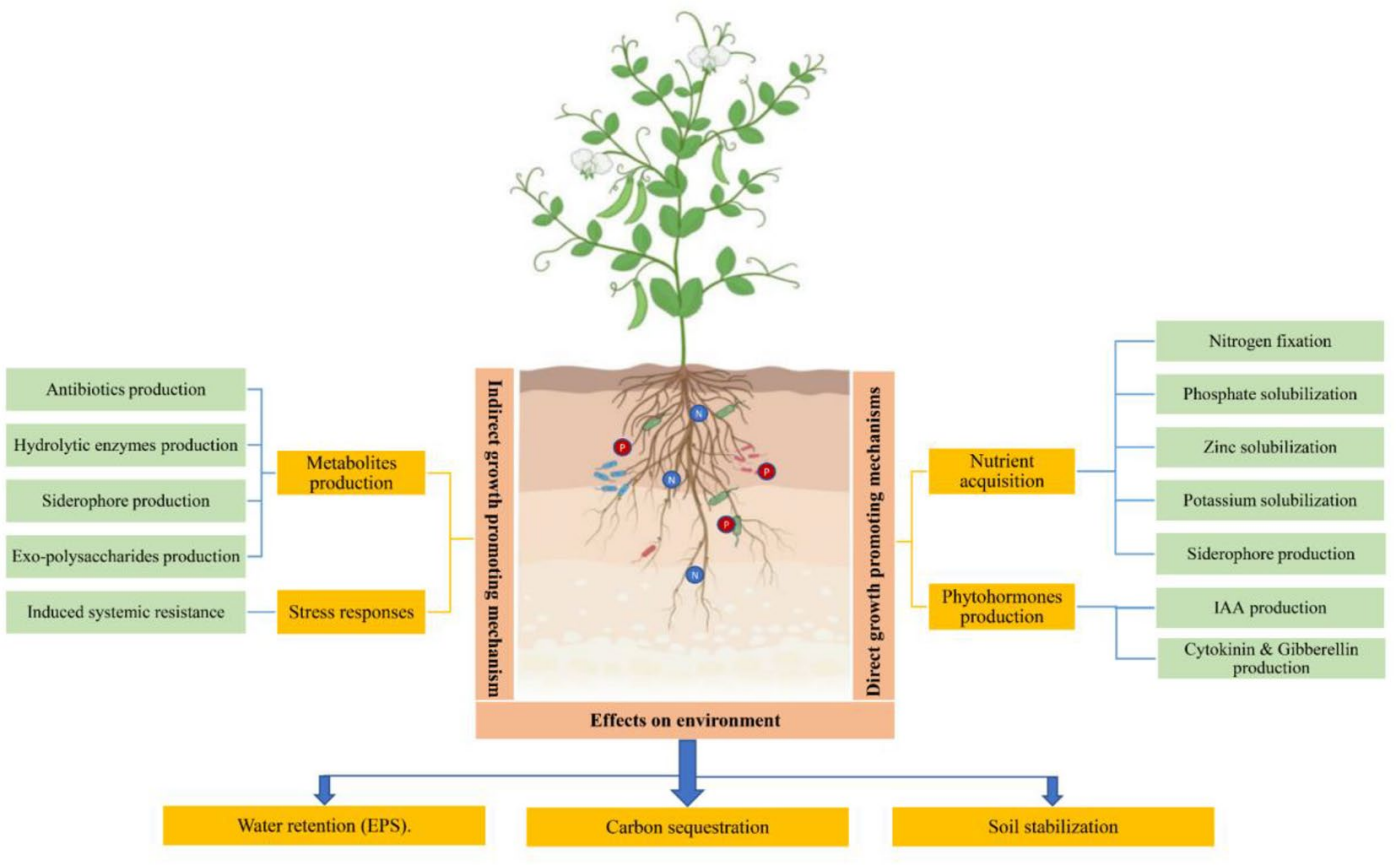
Direct and indirect mechanisms of PGPR in enhancing plant growth and health.

PGPR play a fundamental role in enhancing plant health, both directly and indirectly (Fig. 1). Directly, PGPR enhances crop production and yield by increasing the bioavailability of both micro and macro nutrients and the production of plant growth hormones. Various bacterial strains such as *Bradyrhizobium* spp. and *Azorhizobium* sp fix atmospheric nitrogen into a form that plants can utilize^11^. Other strains including *Bacillus, Rhizobium, and Pseudomonas* are known for solubilizing phosphate, which is crucial for cell division and the development of new tissues in plants^12^. PGPR such as *Agrobacterium, Pseudomonas,* and *Rhizobium* are also involved in the production of plant hormones, such as auxins, that stimulate root growth and development, leading to improved nutrient absorption and overall plant health^13^. On the other hand, PGPR can act as biocontrol agents and indirectly protect plants from various phytopathogens by inducing systemic resistance, a phenomenon where the plant's immune system is primed to respond more effectively to subsequent pathogen attacks. PGPR also produce antimicrobial substances and hydrolytic enzymes such as proteases that inhibit the growth of harmful pathogens in the rhizosphere, creating a healthier environment for the plant's root system^17,18^. Furthermore, rhizobacteria, especially PGPR, play a crucial role in enhancing soil structure, promoting aeration, and improving water retention^19–21^. This vital function contributes significantly to sustainable agriculture by creating a healthier environment for plants to thrive, ensuring optimal nutrient uptake, and ultimately leading to increased crop yields and overall agricultural productivity^22,23^. In addition, PGPR are essential in mitigating the adverse effects of salinity and drought stress on plants^24^. By improving beneficial interactions with plant roots, these bacteria enhance the plant's ability to withstand harsh environmental conditions, making them invaluable in promoting crop resilience in saline and arid regions^25,26^.

Unfortunately, plant pests and diseases inflict significant yield losses, amounting to approximately US$550 billion worldwide annually^27^. While 60% of these losses are attributed to weather, invertebrate pests, and weeds, the remaining 40% are caused by plant diseases, with fungal pathogens accounting for two-thirds of this portion. Annually, fungal plant pathogens destroy over 125 million tons of crucial crops like wheat, rice, potatoes, maize, and soybeans^27^. Currently, the control of phytopathogens heavily relies on agrochemicals, including broad-range fungicides such as Azoxystrobin^28^. However, the environmental impacts associated with the use of agrochemicals such as the loss of biodiversity and pollution due to toxicity and bioaccumulation creates the necessity to find alternative solutions that are more eco-friendly^29^. A favorable substitute for these agrochemicals can be through the application of bio controllers. This method of control employs natural and exotic enemies of pathogens to resist their damage and maintain the health of the plant species^30^. Currently, these biocontrol agents have been increasingly applied in the field of sustainable agriculture and are showing improvements in plant health in different regions around the world^31^.

*Fusarium oxysporum* belongs to the phylum ascomycete which can cause vascular wilts, patch disease, head blights, and root rots in plants. This fungus induces wilt diseases by invading seeds, wounds, and roots and in turn colonizing the xylem tissues of host plants^32^. *F. oxysporum* causes substantial losses in several economically important plant species such as tomatoes and flowers, field crops, such as cotton, and plantation crops, including banana, oil palm, and date palm. Furthermore, controlling this fungus is hard, and using resistant cultivars of plants might be a practical way of controlling the disease in the field. Additionally, crop rotation and fungicide application are used for disease control. In contained spaces such as glasshouses, soil sterilization is performed if feasible^32,33^.

*Botrytis cinerea* is another extensively studied fungal species that can infect more than 200 plant species due to its ability to counteract the plant defense chemicals. This pathogen targets various plant parts including leaves, stems, and fruits of plants such as tomatoes, lettuce, and potatoes^34^. The annual losses due to this fungus range from $10 billion up to $100 billion worldwide^27^. Furthermore, *B. cinerea* uses a broad range of chemicals that could lead to the death of the host plant including oxalic acids, HSTs, and botrydial. In the field, control of grey mold caused by *B. cinerea* usually requires the use of fungicides and reduction of humidity, especially in greenhouses. Additionally, the implementation of antagonistic biocontrol agents holds promising potential for effective control^27^.

Knowledge of the native bacterial populations, their characterization, and identification is essential for understanding the diversity and distribution of indigenous rhizobacteria associated with local plants. Exploration of region-specific microbial strains that could be employed as biocontrol/growth-promoting inoculums to accomplish an anticipated crop production is also very important^35^. This is due to the fact that plant species that face environmental stresses such as high temperatures and salinities might possess PGPR that supports their fitness against these stresses. Several studies showed that PGPR found in arid lands and harsh environments are capable of enhancing the growth of plants as well as withstanding the harsh environment. PGPR such as *Bacillus cereus* and *Bacillus albus* collected from arid lands have unique traits that are compatible with the harsh environmental conditions such as nutrient deficiency, intense solar radiation, high salinity, and drought^36–38^. While biocontrol and bio-pesticide sciences have reached a global stage of commercialization^42^, regional knowledge concerning arid land soil microbiota, particularly in arid regions like Qatar, remains limited^41^. Local studies on PGPR are scarce, and their potential largely goes unrecognized. Compounded by the challenge of food security, particularly in Qatar where vegetables have a short storage time, addressing these gaps becomes crucial. Hence, the primary objective of this research is to explore and investigate local rhizobacteria isolated from Qatari plants aiming to enhance plant growth and reduce reliance on agrochemicals. By exploring the unused resources provided by the unique local ecosystem, this study also aims to contribute significantly to both regional agricultural knowledge, and the promotion of eco-friendly farming techniques in arid lands.

## Materials and methods

### Rhizobacteria collection and isolation

Several field trips were arranged to collect soil and plant root samples. The soil was collected from the rhizosphere zone around healthy plant roots at a depth of around 10–15 cm^43^. The collected samples were kept in labeled plastic bags in an icebox and were brought to the laboratory. Plant root and soil samples from the rhizosphere of 10 plant species were collected at three different sites in Qatar namely: Qatar University biology field-1 (25.371004, 51.491592), Biology field-3 (QUBF1 and QUBF3) (25.370057, 51.495274) and Al-Thakhira area (25.686507, 51.554472). The plant species included in the sampling process were: *Avicennia marina, Suaeda vermiculata, Salsola soda, Anabasis setifera, Salicornia europaea, Arthrocnemum macrostachyum, Limonium axillare, Tetraena qatarensis* (previously known *as Zygophyllum qatarensis)*, *Aeluropus lagopoides, and Prosopis juliflora.*

All the above-mentioned plant species are native to Qatar except *P. juliflora*, which is known as invasive species. Plant root samples were collected after the proper permission and all methods were carried out in accordance with relevant guidelines and regulations. None of the above mentioned plant species are described by IUCN red list as threatening, decreasing or endangered in Qatar.

For soil bacteria, one gram of each soil sample was added to falcon tubes containing 9 ml of sterile distilled water and then centrifuged at 150 rpm for 30 min. The soil suspension was diluted with sterile distilled water to prepare serial dilutions from 10^–1^ to 10^–4^ concentrations. Following that, 0.5 ml of the third and the fourth dilutions were plated on nutrient agar. To collect bacteria from plant roots, the root samples were washed under tap water to remove excess soil and then cut into 2–3 cm pieces using a sterile blade. The surface of each root sample was sterilized using 5% sodium hypochlorite for 1 min^44^. Then the roots were rinsed with sterilized distilled water four times, 10 min each, and then crushed with a sterile mortar and pestle. To isolate the bacterial cells from the roots, one gram of the sample was added to 9 ml of sterile distilled water and agitated in a centrifuge at 150 rpm for 30 min. Bacterial strains capable of laboratory growth were isolated, and individual colonies were subcultured repeatedly on the same agar media to avoid contamination and establish pure rhizobacterial cultures. Following 48 h of incubation at 35 °C, the isolated bacteria were stored in a refrigerator (4 °C) for subsequent use.

### In-vitro investigations for antagonism between the isolated PGPR and plant pathogen

The antagonistic effects of the isolated strains were evaluated using the dual culture method. One 7-mm disc of active mycelia from a pure and freshly prepared culture (5–7 days old) of the pathogens (*F. oxysporum*, and *B. cinerea*) was placed at the center of a Petri dish containing PDA. Using a sterile loop, the isolated rhizobacteria were streaked in a line, 3 cm away from the center of a Petri dish and on both sides^45^. Plates containing only the pathogen were also cultured and considered as a control for fungal growth^41^. All plates were incubated for 5 days for *F. oxysporum*, and 4 days for *B. cinerea* at 25 °C, after that the growth diameter of the pathogen colony (fungal growth) was measured using a digital caliper. Each experiment considering a single bacterial strain was replicated in three plates. The percentage of fungal growth inhibition was calculated according to the following equation:

$$\% {\text{ Inhibition}}\, = \,\left( {{\text{R}}\, - \,{\text{r}}} \right)/{\text{R}}*{1}00$$

where r is the radius of the fungal colony treated with the PGPR and, R is the maximum radius of the fungal colony (control). All isolates were tested in triplicates^45^.

### Identification of isolates using 16 S rRNA sequencing

Due to resource and time limitations, rhizobacterial strains that exhibited significant inhibition of phytopathogens growth in-vitro were identified genetically. DNA extraction and purification were done using a kit (NucleoSpin®). Two universal primers namely RibS73sp: 5′AGAGTTTGATCCTGGCTCAG3′ and RibS74sp: 5′AAGGAGGTGATCCAGCCGCA 3′ were used for the amplification of the 16S rRNA region, and the sequencing of the PCR product was done at external laboratories. Sanger sequencer raw data was read using BioEdit software. Basic Local Alignment Search Tool (BLAST) network services of the National Centre for Biotechnology Information (NCBI) database were used to compare the obtained sequences to the existing sequences.

Furthermore, the 16S rRNA gene sequences of the collected bacterial isolates were compared to known sequences listed in NCBI’s GenBank using BLAST. Multiple alignment of the nucleotide sequences was performed using MUSCLE. The evolutionary history was inferred by using the Maximum Likelihood method and Kimura 2-parameter model in MEGA11. The bootstrap consensus tree was inferred from 1000 replicates representing the evolutionary history of the analyzed data. The percentage of trees in which the associated taxa clustered together is shown next to the branches. The tree is drawn to scale, with branch lengths measured in the number of substitutions per site. This analysis involved 16S rRNA nucleotide sequences of the isolated stains and several other regional and international strains for comparison. The 16S rRNA gene sequence of *Pseudomonas putida* was used as an out-group species^40,46^.

### Bioassays to evaluate growth promoting and biocontrol potential of the isolates

After in-vitro screening, rhizobacterial species displaying significant antagonistic effects against plant pathogens were further evaluated in the presence of the host plant, tomato (*Solanum lycopersicum* L*.*, SC 2121, AGRI-QATAR). Two different experiments were conducted using selected tomato cultivar:

#### Pot experiment design

Seeds of Tomato (*S. lycopersicum* L*.*, SC 2121) were bought from a local market, washed vigorously with tap water, and then air-dried. Following that, the seeds were transferred to plastic trays (40 × 20 cm) containing mixed soil (50% regular Qatari soil and 50% Peatmoss) that was previously autoclaved 4 times at 121 °C for 15 min each, for 4 consecutive days. The seeds were left to germinate in growth chamber conditions (around 20 °C and continuous fluorescent lighting). The plants were watered whenever necessary. After 3 weeks, healthy seedlings of almost similar size (around 5–6 cm height) were transplanted in separate small pots (10 cm diameter) and grown under greenhouse conditions^47^. The PGPR treatments were applied to 1-month-old seedlings. The bacteria suspension was prepared by inoculating falcon tubes containing 40 ± 1 ml of nutrient broth with a loop full of selected strains and were placed in a shaker at 175 rpm at 27 °C for 48 h. The falcon tubes were then centrifuged at 4000 rpm for 20 min to separate the bacteria from the media. The bacterial pellet then was suspended in 0.85% NaCl saline solution and its OD was adjusted to 0.5 ± 0.05 at 550 nm corresponding to about 10^7^–10^8^ CFU ml^−1^^48^. The experimental design was Completely Randomized Design (CRD), and the treatment levels were as follows: Control: (1) Untreated plants, no PGPR was applied; (2) Bacteria treated, the plants were treated twice with 5 ml of the prepared solution of PGPR, the first application was on 4 weeks old seedlings and second on 5 weeks old seedlings. Each treatment level had four replicates. The plants were maintained in the greenhouse and checked regularly. No fertilizer or pesticides were used. At harvesting time, 4 weeks post-treatment, all plants were removed from the soil and their roots were gently cleaned from soil particles, and then the plants were cut into two parts, the shoots, and roots. Plant parts were packed separately in paper bags, oven-dried for 72 h at 80 °C, and then dry weights of the shoot and root parts were measured.

#### Hoagland medium design

Local tomato seeds were surface sterilized using 5% NaOCl for 1 min and then washed with sterilized distilled water three times. The washed seeds were placed in sterilized glass Petri dishes containing filter papers topped with Hoagland solution amended with 0.3% agar. The agar was added to prevent the drying of seeds and contamination from continuous watering. The seeds were placed in a growth chamber with continuous lightning and 25 ± 2 °C for 3 days. The germinated seeds were then transferred aseptically into 25mm wide tubes containing 25 ± 2 ml of Hoagland medium with 0.8% agar and were covered with Parafilm to avoid contamination. The tubes were then placed in a growth chamber with 12 h of light, at 26 °C. After ten days of seedling growth (length around 5 cm, with 4–5 leaves), one ml of the selected bacterial suspension (OD = 0.5 ± 1 at 550 nm,) was added on top of the medium. After 5 days of bacterial inoculation, one 7-mm disc of the active mycelia of *F. oxysporum* was added to each tube and then returned to the growth chamber. The experimental design used was CRD, and the treatment levels were as follows: Control: Untreated plants, no PGPR nor *F. oxysporum* applied; Fungus-treated, one active mycelial disc of *F. oxysporum* was placed close to plant roots. Bacteria-Fungus treated, the plantlets were treated first with 1 ml of the prepared solution of PGPR, and after 5 days, the mycelial disc of *F. oxysporum* was placed close to the plant roots. Each treatment level had three replicates. At harvesting time, plants were transferred to a water bath at 45 °C for 1–2 h to dissolve the agar medium, and then plants were removed from the medium and washed with distilled water. The plants were placed between two tissue papers with gentle pressure to remove excess water from the plant surface. Subsequently, each plant was dissected to separate roots from aboveground shoots, and each part was individually packed in small paper bags, appropriately labeled. These bags were then transferred to an oven and dried at 80 °C for 72 h. The dry weights of both shoot and root parts of each plant were measured.

### Assessment of the potential plant growth promoting and stress tolerance traits of selected strains

Selected isolates exhibiting high effectiveness against plant pathogens underwent various characterization tests to identify their beneficial traits for plants. These tests were conducted in duplicate to qualitatively determine the mechanisms possessed by the PGPR that benefit plants.

#### Hydrogen cyanide (HCN) production test

HCN production of bacterial isolates was tested qualitatively. The selected antagonistic bacteria were grown on King’s B medium amended with glycine at 4.4g/l. Following that, sterile filter paper saturated with a picric acid solution (2.5 g of picric acid; 12.5 g of Na_2_CO_3_, 1000 ml of distilled water) was placed in the upper lid of the Petri plate. The selected isolates were plated, and the dishes were sealed with Parafilm and incubated at 28 °C for 4 days. A change of color of the filter paper from yellow to brown, or reddish-brown was recorded^49^.

#### Phosphate solubilization activity test

Qualitative estimation of phosphate solubilization was done using the National Botanical Research Institute's phosphate growth medium (NBRIP) that contained the following (per liter): 10g Glucose, 5 g Ca_3_(PO_4_)_2_, 5 g MgCl_2_.6H_2_O, 0.25 g MgSO_4_.7H_2_O, 0.2 g KCl, 0.1 g (NH_4_)_2_SO_4_^50^. Plates were inoculated in duplicates with the selected bacterial strains.

#### Hydrolytic enzyme production tests

The selected strains were also tested for two hydrolytic enzyme production capabilities using two different mediums. To determine the protease activity, the isolates were cultured on skim milk agar and kept at 30 °C for 48 h. The formation of a clear zone around the bacteria indicated the production of proteases. On the other hand, to determine the cellulase activity, isolates were stroked on a minimal medium amended with carboxymethyl cellulose (CMC) as a carbon source (Composition in g/L: KH_2_PO_4_, 2.0; (NH_4_)_2_SO_4_, 1.4; MgSO_4_·7H_2_O, 0.3; CaCl_2_, 0.3; FeSO_4_·7H_2_O, 0.005; MnSO_4_, 0.0016; ZnCl_2_, 0.0017; CoCl_2_, 0.002; CMC-Na, 10.; and agar; 15). The plates were incubated at 30 °C for 72 h. After that, plates were flooded with 0.1% Congo red solution for 20 min and then washed with 0.1M NaCl solution. The presence of a clear zone around the colony indicated cellulase activity^46,51^.

#### Salinity tolerance tests

The selected rhizobacteria were subjected to salinity tolerance testing by streaking them onto nutrient agar plates containing 5% and 10% NaCl. Subsequently, these plates were incubated at 30 °C for 48 h to observe the bacterial growth and response to different salinity levels^52^.

#### Qualitative estimation of ACC Deaminase activity

To test for ACC deaminase activity, selected rhizobacteria were plated on sterile minimal DF salts media containing the following (per liter): 2.0 g glucose, 4 g KH_2_PO_4_, 0.2 g MgSO_4_.7H_2_O 6 g Na_2_HPO_4_, 2.0 g citric acid, 10 mg H_3_BO_3_,1 mg FeSO_4_.7H_2_O, 124.6 mg ZnSO_4_.7H_2_O, 11.19 mg MnSO_4_.H_2_O, 10 mg MoO_3_, 78.22 mg CuSO_4_.5H_2_O. 3 mM ACC was added as a sole source of nitrogen instead of (NH_4_)_2_SO_4_. The pH of the media was adjusted to 7.2 and supplemented with 1.5% agar. The cultures were incubated at 28 °C for 3 days and growth was monitored daily. Bacteria growing on the plates were considered ACC deaminase producers^53^.

#### IAA production test

Rhizobacteria were also tested for their ability to produce IAA. Bacterial cultures were grown in an LB medium supplemented with 2g/l L-tryptophan. After 4 days of incubation at 37 °C, the cultures were centrifuged at 4000 rpm for 30 min, following that 1 ml of supernatant was mixed with 2ml of Salkowaski reagent, and the tubes were kept in the dark for 30 min, the formation of pink color indicated IAA production^54,55^.

#### Ammonia production test

The selected rhizobacteria were evaluated for ammonia (NH_3_) production by culturing the isolates in peptone broth at 30 °C for 7 days. Following incubation, 0.5 ml of Nessler’s reagent was added, and the development of a yellow–brown color indicated the production of NH_3_^56,57^.

### Statistical analysis

For greenhouse and growth chamber experiments, the experimental design used was a Completely Randomized Design (CRD). One-way ANOVA followed by Tukey Post-Hoc test was used to evaluate the significance of the measured parameters at *p* ≤ 0.05. For in-vitro antagonism data, results were expressed as means and standard deviations (S.D.) for percentage inhibition of *F. oxysporum* and *B. cinerea* growth in the presence of each rhizobacterial isolate.

## Results & discussions

### Collection of isolates

During five field trips and three different locations in Qatar, a total of 55 bacterial isolates were obtained from the rhizosphere of 10 different plant species. The number of bacterial isolates obtained from each plant species is presented in (Table 1). Qatar’s habitat is characterized by elevated temperatures (max ~ 49 °C), salinity, and scarce rainfall (average ~ 78.1 mm). These meteorological conditions have strong effects on the terrestrial environment in terms of soil properties. The soil is sandy-sandy clay loam with a pH range of (6.6–8.4) and an absolute water content % of (2.2–25.7). Therefore, many of the coastal area plants as well as the inland plants are considered halophytes due to their anatomical, morphological, and physiological adaptation to the local environment^58^.

**Table 1:** Summary of the number of isolates collected from the rhizosphere of selected plant species.

Plant Species	Number of isolates	
	Roots	Soil
*A. lagopoides*	3	–
*A. setifera*	4	1
*A. macrostachyum*	4	–
*A. marina*	6	3
*L. axillare*	3	–
*P. juliflora*	3	2
*S. europaea*	8	–
*S. soda*	5	–
*S. vermiculata*	3	5
*Tetraena qatarensi*	5	–

### In-vitro investigations for antagonism between the isolated rhizobacteria and the plant pathogens

**Figure 2:**
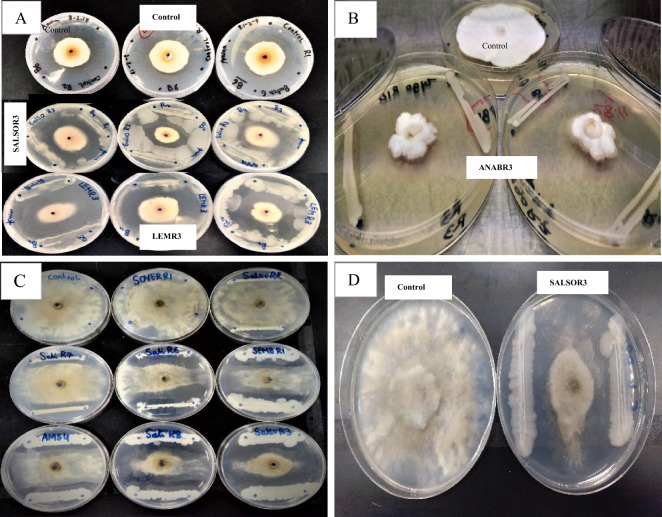
(**A**, **B**) Images showing the effects of isolated rhizobacteria on growth of *F. oxysporum* and (**C**, **D**) *B. cinerea* in-vitro.

The antagonistic abilities of the rhizobacterial isolates against two significant plant phytopathogens, *F. oxysporum* and *B. cinerea*, were investigated using a dual culture technique. It should be realized that for *F. oxysporum,* the inhibition % was recorded after 5 days of culturing, while for *B. cinerea,* it was recorded after 4 days, due to its faster growth (Table 2, Fig. 2).

**Table 2:** Percentage of growth inhibition (mean values and standard deviations S.D.) of *F. oxysporum* and *B. cinerea* caused by each of the 55 isolates of bacteria collected from the rhizosphere of Qatari flora.

#	Strain Code	Average Inhibition (%) (*F. oxysporum*)	SD	Average Inhibition (%) (*B. cinerea*)	SD
1	AELUR1	21.70	2.03	30.94	5.16
2	AELUR2	30.73	13.39	43.48	1.19
3	AELUR3	16.56	3.98	14.49	7.52
4	AMRR1	− 7.53	5.38	− 1.08	3.50
5	AMRR2	− 6.83	1.58	53.63	2.14
6	AMRR3	2.12	4.08	46.15	6.96
7	AMRR4	13.27	17.11	49.10	2.22
8	AMRR5	13.67	20.62	45.26	4.89
9	AMRR6	25.46	14.95	48.23	3.45
10	AMRS1	4.94	4.75	9.88	4.69
11	AMRS3	23.28	16.18	34.54	2.78
12	AMRS4	5.12	18.44	10.24	3.96
13	ANABR1	18.25	6.76	42.27	0.53
14	ANABR2	7.13	10.31	43.73	1.87
15	ANABR3	37.89	27.07	38.51	0.49
16	ANABR4	25.78	17.20	43.29	1.14
17	ANABS1	21.63	8.68	9.38	13.98
18	ARTHAR1	19.56	8.04	45.63	4.69
19	ARTHAR2	18.54	5.51	42.12	2.11
20	ARTHAR3	29.73	5.77	52.71	2.56
21	ARTHAR4	− 27.66	12.21	4.60	4.24
22	LEMR1	10.36	9.66	39.49	6.51
23	LEMR2	5.75	8.41	23.38	7.32
24	LEMR3	36.30	15.81	55.60	6.39
25	PROSON1	10.00	7.64	28.03	0.57
26	PROSON2	10.39	8.37	5.08	3.93
27	PROSON3	5.54	12.39	29.86	2.19
28	PROSOS1	13.06	19.62	0.30	2.44
29	PROSOS2	7.82	4.57	− 3.32	0.84
30	SALIR1	48.73	7.12	21.34	8.72
31	SALIR2	9.22	11.69	48.61	0.98
32	SALIR3	− 0.07	2.85	0.98	5.66
33	SALIR4	16.58	10.19	− 1.69	2.06
34	SALIR5	49.06	4.25	21.01	4.91
35	SALIR6	− 8.01	20.16	44.63	4.66
36	SALIR7	23.41	21.61	40.07	1.21
37	SALIR8	9.32	5.84	56.25	4.92
38	SALSOR1	18.46	16.64	1.84	2.10
39	SALSOR2	34.86	2.06	39.68	2.68
40	SALSOR3	17.32	4.84	57.19	2.93
41	SALSOR4	6.53	4.78	− 0.96	0.51
42	SALSOR5	− 15.21	2.40	27.52	0.67
43	SOVERR1	− 24.79	9.54	− 0.80	2.09
44	SOVERR2	15.91	10.48	55.84	0.44
45	SOVERR3	− 4.54	4.16	6.76	2.64
46	SOVERS1	− 13.92	4.82	25.35	1.83
47	SOVERS2	8.57	9.57	53.05	0.91
48	SOVERS3	14.80	15.85	47.65	1.10
49	SOVERS4	17.84	19.82	29.15	3.01
50	SOVERS5	34.45	19.66	42.56	2.14
51	ZYGOR1	− 7.31	5.03	3.21	2.68
52	ZYGOR10	31.91	9.91	30.62	2.95
53	ZYGOR7	28.11	6.32	32.22	1.20
54	ZYGOR8	− 8.10	0.53	4.89	7.13
55	ZYGOR9	− 1.95	14.03	0.29	1.51

The isolated rhizobacteria exhibited varying degrees of inhibition against *F. oxysporum*, ranging from − 27.66% (ARTHAR4) to a remarkable maximum inhibition of 49.06% (SALIR5). Notably, several isolates displayed strong antagonistic activities, inhibiting *F. oxysporum* growth by 28–49.06%, with an average inhibition rate of approximately 12.61%. Intriguingly, only two of these isolates managed to suppress the fungal growth by nearly 50%. No clear pattern emerged regarding strong inhibition from specific plant isolates or particular collection sites. It was evident that rhizobacteria with varying antagonistic abilities were present across different plants and locations. The highest inhibition of *F. oxysporum*, at 49.06%, was achieved by isolate SALIR5, collected from the Al-Thakhira area. Notably, among the top 10 isolates with the highest antagonist percentage, a majority were sourced from Al-Thakhira. However, this observation could be attributed to the more frequent sampling conducted in this location compared to the other two sites. Our results about isolating effective rhizobacterial strains from the Qatari environment almost coincide with results obtained from another study accomplished in Saudi Arabia^41^, a country that has almost similar environmental conditions. The authors isolated more than 500 rhizobacterial strains from 11 different wild plants in Almadinah Almunawarah, and they reported that the in-vitro growth inhibition of *F. oxysporum* ranged from 30% up to 70%. In the same study, they also tested the rhizobacteria against another phytopathogen, *i.e., Sclerotinia sclerotiorum*, authors stated that in general, the effect on *F. oxysporum* was lower^41^. In another study by Recep et al^59^., PGPR were tested against *Fusarium* species. In-vivo Petri-plate tests showed the collected rhizobacteria had an inhibitory effect ranging from 3 to 45% against *F. oxysporum*. The maximum inhibition was caused by *Burkholderia cepacia*.

On the other hand, the results of the dual culture test against *B. cinerea* showed that the inhibition percentage ranged from − 3.32% (PROSOS2) up to 57.19% (SALSOR3) with an average of about 29.12% (Table 2). The inhibition percentage of various rhizobacteria and the average inhibition is notably higher compared to those of *F. oxysporum.* Similarly, in the case of *F. oxysporum*, no specific plants harbored strong antagonists of *B. cinerea.* However, it should be noticed that inhibition was generally stronger against *B. cinerea,* for example, in the case of strain (LEMR3) the growth inhibition of *F. oxysporum* was found to be (36.3%) whereas the same isolate caused 55.60% inhibition for *B. cinerea* (Table 2). Similar cases can be noticed in many other strains. In the case of *F. oxysporum,* 12 strains of the 55 (around 21% of the collected strains) did not show any growth inhibition signs, while the remaining 43 had a range of effects. For *B. cinerea,* only five strains (10% of the whole collection) did not show any in-vitro antagonism. These facts also support the idea that collected rhizobacteria had a stronger effect on *B. cinerea* compared to *F. oxysporum* coinciding with the study done in Saudi Arabia as mentioned earlier^41^*.* In another study by Zdravković et al^60^., the effects of *Pseudomonas* spp. and *Bacillus* sp. on *B. cinerea* were tested in-vitro. It was found that these rhizobacteria had a range of effects from around 39 to 80% inhibition of fungi growth after 7 days of incubation. In the studies mentioned, several bacterial isolates displaying significant antagonistic activities against prevalent plant pathogens were identified, particularly in the arid lands of the Arabian Gulf region (Table 5). For instance, *Streptomyces* species from Qassim region in KSA exhibited antagonism against *Colletotrichum gloeosporides* and *Alternaria solani*^61^. Similarly, *Bacillus, Enterobacter,* and *Pseudomonas* species showed strong antagonism against *F. oxysporum* and *S. sclerotiorum*^41^*.* Additionally, endophytes like *Cronobacter muytjensii* from Jizan-KSA inhibited the growth of the phytopathogenic *Phytophthora infestans*^62^. These findings underline the diverse and potent antagonistic capabilities of rhizobacteria from the arid lands of the Arabian Gulf against various plant pathogens, highlighting their potential for biocontrol strategies in the harsh environmental conditions of these regions.

### Identification of selected rhizobacterial strains

**Figure 3:**
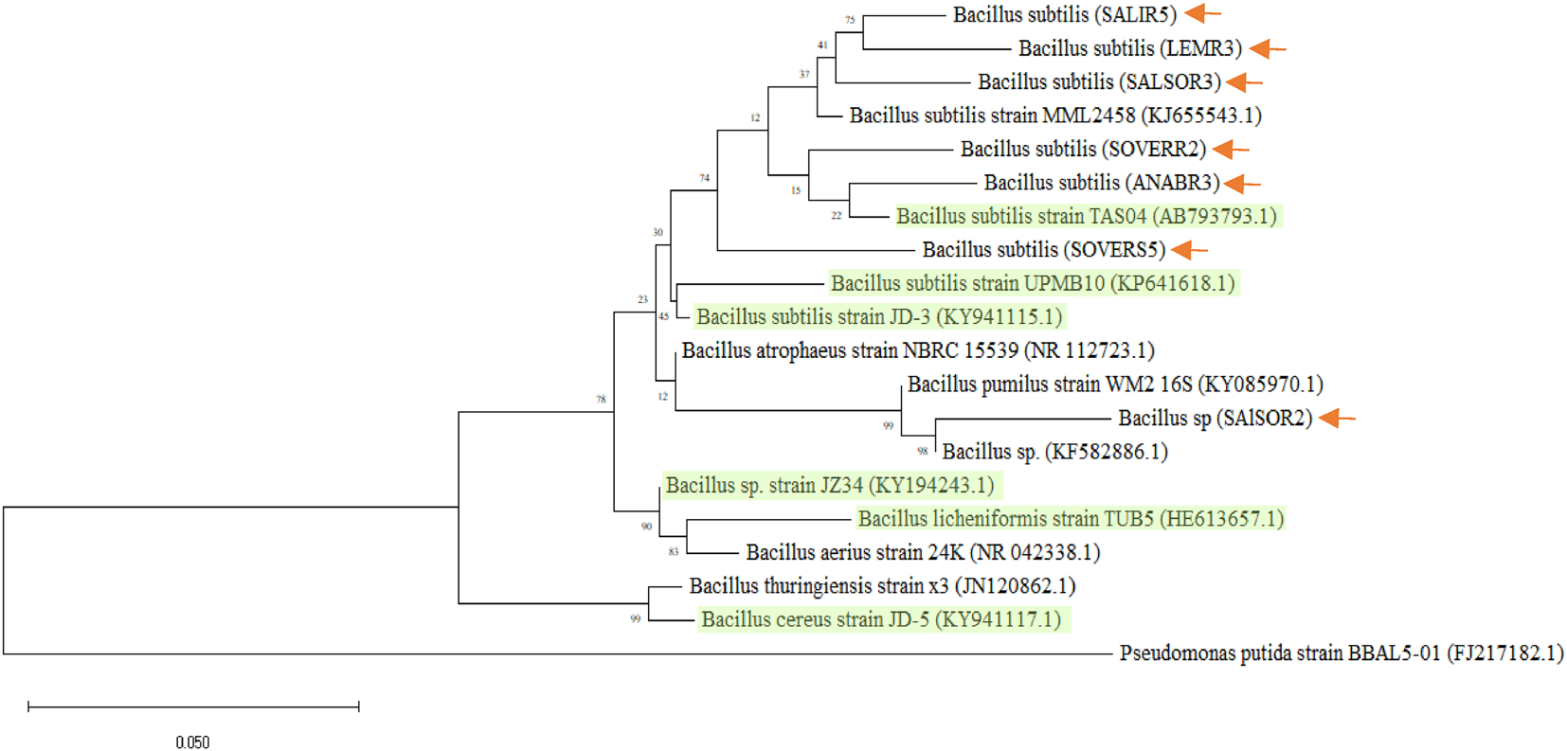
Phylogenetic tree showing the relationship between the local rhizobacterial isolates (indicated by the arrows) and some examples of rhizobacterial collected the Arabian Gulf (highlighted) based on the 16S rRNA gene sequences. The 16S rRNA sequence of P. putida Strain BBL5-01 was used to assign an outgroup species. The scale bar indicates 0.05 substitutions per nucleotide position.

According to top Blast similarity matches, the 16S rRNA gene sequence analysis and the phylogenetic analysis, all the rhizobacterial isolates belonged to the genus *Bacillus* (Table 3 and Fig. 3). These results show that *Bacillus* strains were diverse at the species level yet have shown more similarities to *B. subtilis* stains MML2458 and TAS04. Numerous studies have consistently demonstrated that the cultivable rhizobacterial population in the rhizosphere of various plants, such as rice, wheat, and tobacco, is predominantly composed of the *Bacillus* genera^46^.Bacteria belonging to this genus are microorganisms that retain extraordinary features that contribute to their importance as plant growth-promoting rhizobacteria and biocontrol agents. These microorganisms are capable of nitrogen fixation, IAA production, and phosphate solubilization thus directly promoting the health of the associated plants (Table 5). On the other hand, *Bacillus* species are also capable of producing antibiotics, siderophores, HCN, and hydrolytic enzymes, all of these contribute to their bio-control abilities^63–65^.

**Table 3:** Results of 16S ribosomal RNA sequencing for selected strains.

Code	Strain	% Similarity	Accession #
LEMR3	*B. subtilis*	98	KJ655543.1
SALIR5	*B. subtilis*	97	KJ655543.1
SOVERR2	*B. subtilis*	97	MG738313.1
SOVERS5	*B. subtilis*	96	MN745903.1
SALSOR2	*Bacillus sp.*	96	KF582886.1
SALSOR3	*B. subtilis*	98	KM192350.1
ANABR3	*B. subtilis*	96	MK629788.1

In the current study, bacterial characterization revealed that all collected strains are Gram-positive rods, contrasting with the findings of Antoun^66^, who observed mostly Gram-negative rhizobacteria. This discrepancy may be attributed to the harsh local conditions, where Gram-positive bacteria are better suited to withstand high temperatures, drought, and salinity without losing viability. Supporting this observation, most isolates were capable of endospore formation, a crucial adaptation for survival in challenging environments like those found in Qatar and the Arabian Gulf region. This resilience is especially significant given the arid conditions in this area, as highlighted in studies by Islam et al^44^. in Bangladesh, where similar Gram-positive motile rods were observed commonly. Additionally, a study in Saudi Arabia (KSA) also identified *B. subtilis* (Gram-positive) as a prevalent rhizobacterial species^41^. In this study, bacterial mobility was also observed in a few strains during microscopic examination. It is important to note that bacterial motility is a significant feature that plays a substantial role in competition and the root colonization process^44^.

*B. subtilis* is a rod-shaped, gram-positive, spore-forming, competitive, motile bacterial species, these bacteria are remarkably diverse and are capable of growth within many environments including harsh arid land environments^65^. Due to their ability to form dormant endospores, *B. subtilis* can persist through drought, nutrient deprivation, and other environmental stresses such as salinity and elevated temperatures^67^. Due to these robust characteristics *B. subtilis* is being increasingly used and studied to enhance plant growth and agricultural productivity^68,69^. Studies have consistently shown that *Bacillus* species, including *B. subtilis*, dominate the rhizobacterial populations in arid land plants, underlining their prevalence and importance in these environments (Table 5)^41,70^.

### In-vivo greenhouse evaluation of tomato seedlings growth promotion

Numerous studies suggest that variation in the effectiveness of rhizobacteria against phytopathogens could occur due to environmental conditions as well as competition between other microorganisms present in the rhizosphere^71^. Thus, it is important to perform in-vivo studies to determine if the rhizobacteria would act similarly to in-vitro experiments in terms of pathogen inhibition, and growth promotion.

**Figure 4:**
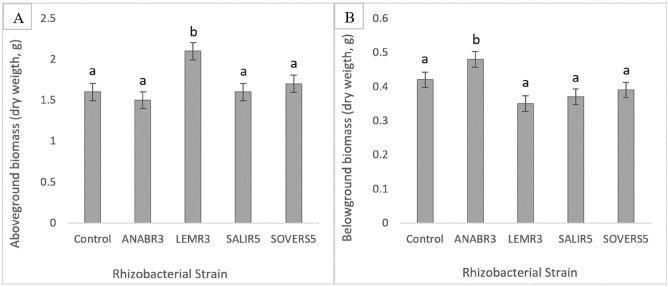
(**A**) Effect of the potential rhizobacterial strains on aboveground biomass of tomato plants at 3 weeks post-treatment (**B**) the effects of PGPR on belowground biomass of tomato plants. Error bars represent the standard error of the mean.

Based on the initial evaluation, several strains were chosen for a detailed evaluation of growth promotion in tomato seedlings under greenhouse conditions due to time limitations and similarities in the results obtained from the antagonistic in-vitro studies. Four week-old tomato seedlings were treated with different rhizobacterial strains and three weeks post-treatment, the plants were harvested and the dry weights of above and below-ground biomasses were measured (Fig. 4). It was found that LEMR3 had significantly increased (*p* ≤ *0.05*) the above-ground biomass of the seedlings by 26.4% compared to the control. Whereas for belowground biomass, it was found that ANABR3 exerted significant effects of biomass increase by 14.6% compared to control plants. However, other strains had no significant effect on the foliage and root growth of tomato seedlings. From the greenhouse in-vivo trials, it could be concluded that these rhizobacteria (LEMR3 and ANABR3) might possess one or more plant growth-promoting properties. The growth-promoting effects are similar to a study in which *Bacillus* and *Pseudomonas* species were found to increase the biomass of tomato seedlings compared to the untreated controls under greenhouse conditions. In this study, the authors stated that the investigated rhizobacteria exhibited PGP traits including IAA and phosphate solubilization^47^. Goswami et al^70^., also studied the effects of PGPR in pot experiments. The results revealed significant improvements in various growth parameters of peanut plants treated with the rhizobacterial strain *Bacillus licheniformis*. The study reported remarkable increases, including a 31% rise in total plant length, a 39% increase in root length, a 44% increase in dry biomass, and a 43% rise in fresh biomass^70^. Another study conducted in China explored the impact of *Bacillus amyloliquefaciens* Strain W19 on banana growth in pot experiments. Initially, no significant differences in plant height, biomass, and stem diameter were observed between the treated and control banana seedlings. However, after 60 days of inoculation and growth, the treatments exhibited substantial effects. A remarkable increase of 12.33% in plant height, 21.53% in fresh weight, 34.63% in dry weight, and 21.33% in stem diameter of the banana plants was documented^72^. Lin et al^73^., also mentioned that applying PGPR mixtures with half the recommended quantity of nitrogen fertilizers resulted in enhanced corn growth and yielded corn biomass and tissue nitrogen concentrations equal to or greater than those achieved with the full nitrogen fertilization rate under greenhouse conditions. This highlights the pivotal role these bacteria play in enhancing plant growth, thereby contributing significantly to the advancement of sustainable agricultural practices. Several studies conducted in similar arid regions have explored the impact of specific bacterial strains on plant biomasses (Table 5). In UAE, endophytic actinobacteria from *A. marina*, specifically *Streptomyces mutabilis*, were found to enhance biomass (dry weight) and increase root length, accompanied by elevated levels of auxins and cytokinins, along with a decrease in abscisic acid^74^. Similarly, in the Red Sea coast region, endophytic bacteria such as *Actinobacteria, Proteobacteria, Bacillus* sp collected from various plants, including *T. terrestris, Z. simplex,* and *P. turgidum*, demonstrated substantial in-vivo effects leading to increase in both shoot and root weight under salt stress conditions^37^. These studies collectively demonstrate the diverse roles of specific bacterial strains in promoting plant growth and stress tolerance, offering valuable insights into sustainable agricultural practices in challenging environments.

### In-vivo Hoagland medium experiment and evaluation of antagonism against *F. oxysporum*

**Figure 5:**
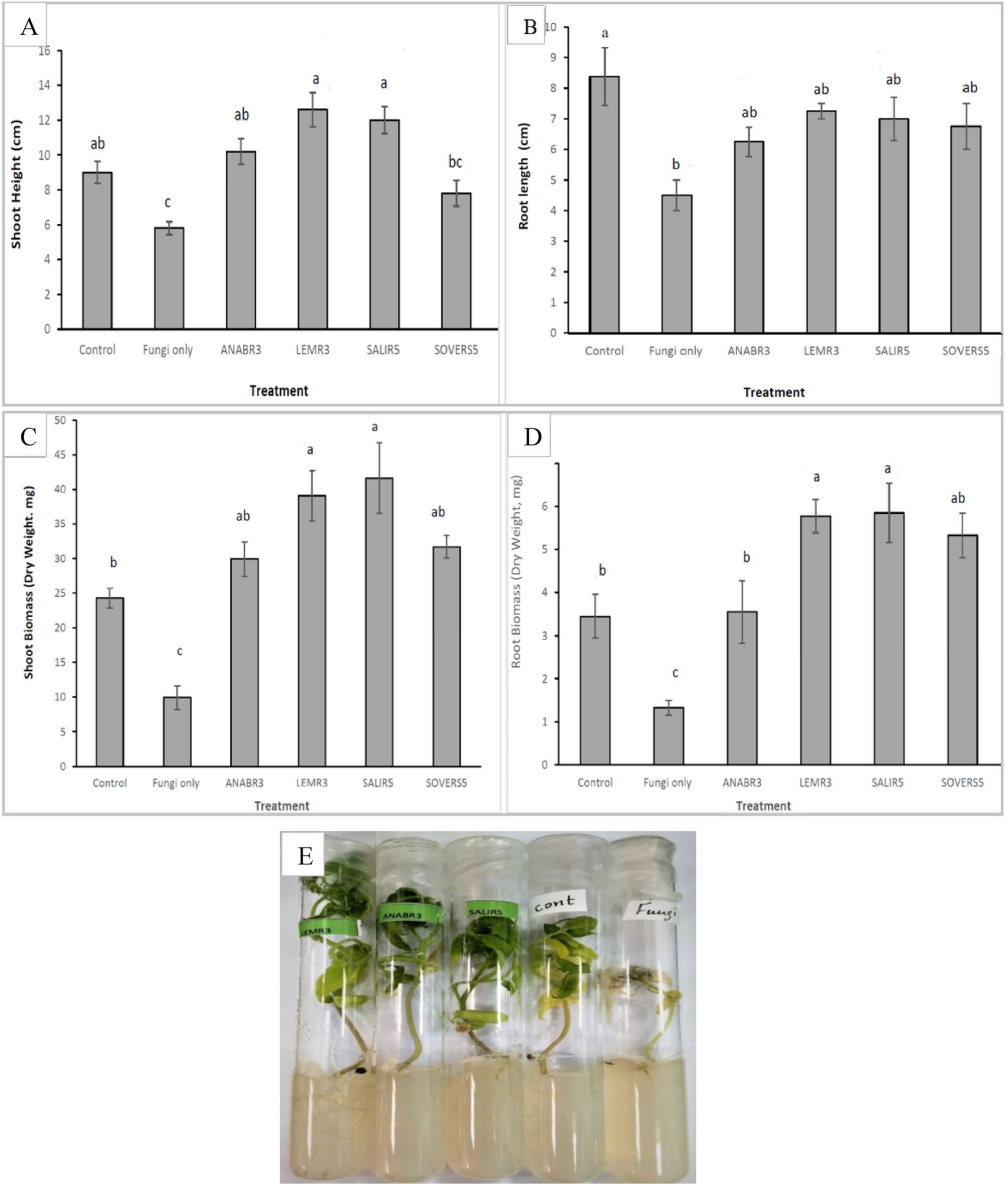
Tomato seedlings (**A**) shoot heights, (**B**) root length, (**C**) shoot dry weight (**D**) and root biomass as influenced by different treatments of either plant pathogen (*F. oxysporum*) and different rhizobacterial strains compared to the control treatment. According to Tukey’s test, mean values having the same letters show no significant difference at *p* ≤ 0.05. Error bars represent the standard error of the means (n = 4), (**E**) In-vivo effects of selected rhizobacterial isolates on tomato seedlings infected with *F. oxysporum* under growth chamber conditions.

This experiment was conducted to evaluate the antagonistic effects of selected rhizobacterial isolates against *F. oxysporum* in the host plant (tomato). *F. oxysporum* was specifically chosen due to its resistance compared to *B. cinerea* as noticed in the in-vitro studies. After three weeks of treatment, the plants were removed from tubes and their shoot and root lengths, and dry biomasses were recorded. It was found that the selected rhizobacteria had a variable effect on the shoot and root biomasses and plant heights. Tomato seedlings treated with *F. oxysporum* alone were significantly reduced (*p* ≤ 0.05) in heights and biomass compared to control and all other seedlings treated with the rhizobacteria (Fig. 5).

In the presence of both rhizobacterial strains and the phytopathogen, tomato seedlings successfully overcame the disease, displaying no significant impact on plant height or the growth of roots and biomasses across all replicates. Notably, none of the tomato seedlings, except those treated with the fungus alone, showed any evidence of disease symptoms. The in-vivo tests revealed significant effects on shoot and root dry biomasses and lengths. In this experiment, all the strains significantly influenced both shoot and root dry weights compared to the treatment with the fungus alone. With the exception of ANABR3 in terms of root dry biomass, all strains exhibited a notable increase compared to the control group (untreated plants). Particularly noteworthy were the effects observed with strains LEMR3 and SALIR5, which exerted significant influences (*p* ≤ 0.05) on shoot height, above, and below-ground biomasses compared to untreated controls as well as the fungi treated seedlings (Fig. 5). These results and the results of greenhouse studies indicate that LEMR3 significantly enhanced the growth of the host plant in the presence and absence of the phytopathogen, underscoring its high potential as a PGPR.

In a similar study done in KSA, PGPR mainly *Pseduomonas aeruginosa* and *B. amyloliquefaciens* significantly reduced the incidence of damping-off disease of cucumber and showed antagonistic effect against the pathogenic *Pythium aphanidermatum* under greenhouse conditions. The authors also stated that in the absence of phytopathogen, these PGPR significantly improved plant biomasses^75^. In another study done in Turkey, the antagonist effects of fluorescent *Pseudomonas* sp. and other microorganisms were tested against *F. oxysporum* in a greenhouse pot experiment^76^. It was found that the disease incidence reduction in tomato plants ranged from 30 to 70%, and the highest reduction in disease occurred when *T. harzianum* T-22 and fluorescent *Pseudomonas* species were used in combination^76^. Wang et al^72^. tested *B. amyloliquefaciens* in the presence of *F. oxysporum* in field trials and found that the incidence of *Fusarium* wilt disease was significantly repressed by around 43% when treated with *B. amyloliquefaciens* strain W19. This study also confirmed that with time, the effects of the treatment were becoming more substantial in both plant growth promotion and disease suppression^72^. Based on the in-vivo studies, it is clear that there is a pressing need for extensive field studies involving larger groups of isolates and different types of microorganisms to comprehensively assess their real-world effects and further enhance our understanding of their potential applications in agriculture.

### Assessment of plant growth promoting and stress tolerance traits of bacterial isolates

**Figure 6:**
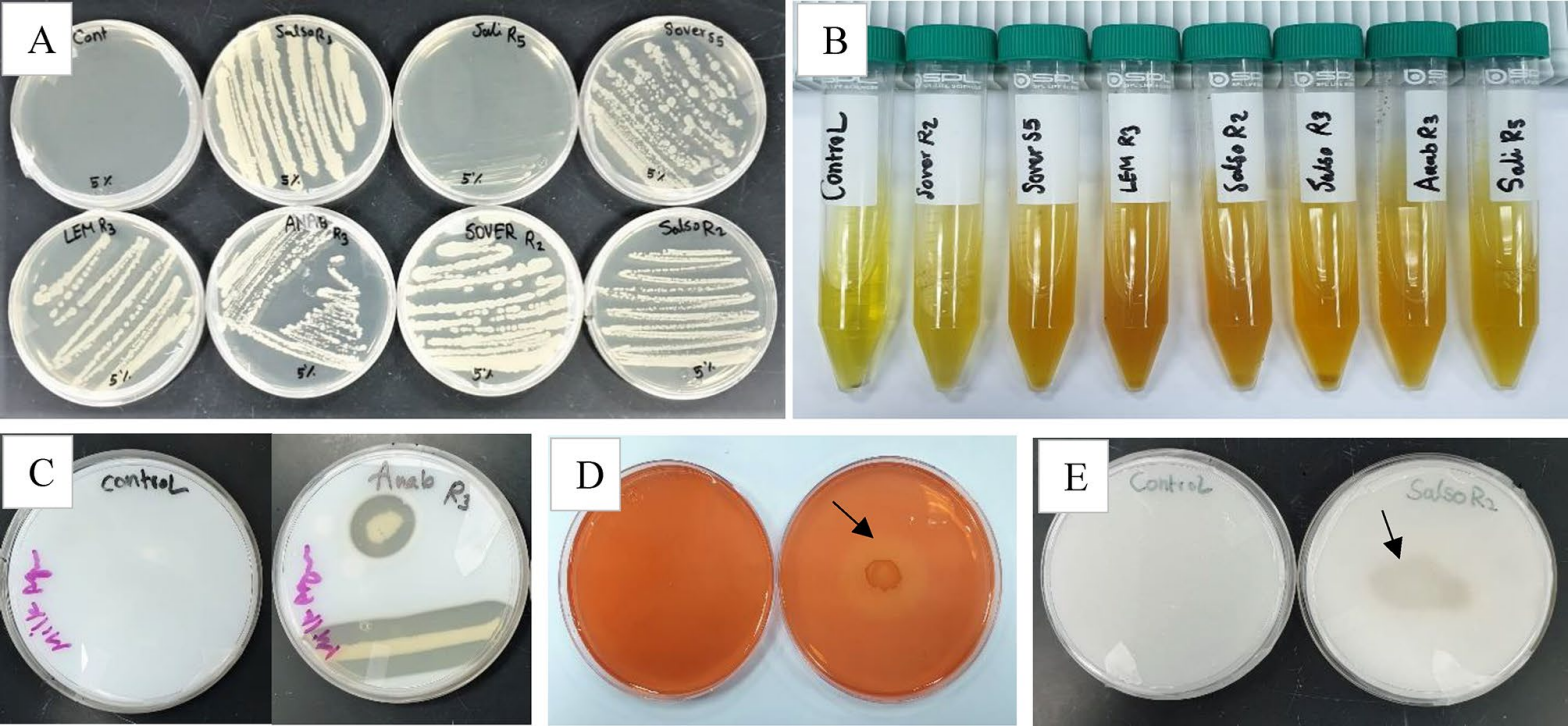
Examples of PGP and stress tolerance traits of selected isolates in terms of: (**A**) Tolerance to salinity 5%, (**B**) Ammonia production, (**C**) Protease activity, (**D**) Cellulase activity (**E**), and Phosphate solubilization.

Following the dual culture testing, selected rhizobacteria were investigated for their plant growth-promoting traits. The results are summarized in (Table 4) and (Fig. 6).

**Table 4:** Assessment PGP and stress tolerance traits of isolated rhizobacteria.

Strain ID	Species name	HCN	Phosphate	Hydrolytic enzymes		Salinity		ACC deaminase	IAA	NH_3_
				Protease	Cellulase	5%	10%			
LEMR3	*B. subtilis*	–	Weak	+	+	+	+	+	+	+
SALIR5	*B. subtilis*	+	–	Weak	–	Weak	–	–	+	Weak
SOVERR2	*B. subtilis*	**–**	–	+	+	+	Weak	+	–	Weak
SOVERS5	*B. subtilis*	**–**	+	+	+	+	+	+	–	+
SALSOR2	*Bacillus sp.*	**–**	**–**	+	+	+	+	+	+	+
SALSOR3	*B. subtilis*	–	Weak	+	+	+	Weak	+	–	+
ANABR3	*B. subtilis*	+	+	+	+	+	Weak	+	–	+

Several isolates produced HCN as can be seen from the change in color from yellow to reddish brown. Many studies confirmed that *Bacillus* species including *B. subtilis* are capable of producing HCN and can positively affect the health of the plant^64,77^. Even though initially HCN production was considered as a biocontrol mechanism against phytopathogens^78^, yet, more recent studies suggested that HCN could be toxic to plants and can lead to plant growth inhibition^79^. However, in oligotrophic environments, HCN might be beneficial to plants by iron sequestration and thus preventing the formation of complexes with phosphates, therefore, increasing the availability of phosphates for plants^80^.

Phosphate solubilization is another important feature of rhizobacteria that directly improves the growth of plants. In the current study, strains SOVERS5 and ANABR3 were found to solubilize phosphate in a solid medium. It was observed that ANABR3 also improved the growth of plants in the greenhouse bioassay, in the absence of phytopathogen, this could be due to the ability of these bacteria to directly increase the availability of nutrients in the rhizosphere of the host plant. Unlike bacteria, plants can only utilize mono or dibasic phosphate, thus plants need bacteria to make the organic or insoluble phosphate available to them^81^. Numerous studies have shown that phosphate-solubilizing microorganisms (PSM) include many bacterial species that can dissolve insoluble phosphates such as *Rhizobium*, *Pseudomonas*, and *Bacillus* including *B. subtilis*. Such species use two processes for phosphate dissolving, either through acidification by releasing acids of low molecular weights like gluconic acid that chelates the positive cations attached to the phosphate molecules or by the production of phosphatases and phytases which hydrolyze the compounds containing organic forms of phosphate^82–85^. The use of phosphate-solubilizing bacteria as bio-fertilizing inoculates has been proven beneficial in increasing yields^81^ and several studies in the Arabian Gulf have mentioned that rhizobacteria were capable of phosphate solubilization (Table 5).

**Table 5:** PGPR studies conducted in the Arabian Gulf region.

Sampling location	Source of PGPR	Examples of identified species	PGP activities	References
Qatar	Rhizobacteria from the rhizosphere of various desert plants	*Bacillus* species including *B. subtilis*	Antagonistic activity against *F. oxysporum* and *B. cinerea,* production of ammonia, IAA, protease, cellulases, HCN, phosphate solubilization and, in-vivo growth promotion of tomato seedlings	This study
KSA- Qassim	Soil samples were collected from the rhizosphere area of healthy plants	*Streptomyces cinereoruber, Priestia megaterium, Rossellomorea aquimaris, and Pseudomonas plecoglossicida*	Phosphate solubilization, production of IAA and siderophore	^43^
KSA- Qassim	Soil samples were collected from agricultural soil around healthy plants	*Streptomyces* spp	Production of siderophores, IAA, and phosphate solubilization, antagonism against the *C. gloeosporides* and *A. solani*	^61^
UAE	Endophytic actinobacteria collected from *A. marina*	*S. mutabilis*	Biomass increase of shoots and roots, increased root length, and other growth parameters of Mangrove. Production of auxins, cytokinins, accompanied by a decrease in abscisic acid levels	^74^
KSA-Jizan	Endophyte of native species	*C. muytjensii*	Increase in salt tolerance of *Arabidopsis thaliana*, production of indole, VOCs, and growth inhibition of two strains of the phytopathogenic oomycete *Phytophthora infestans*	^62^
KSA	Endophytes of *Zygophyllum simplex*	*Paenibacillus sp.*	Promote salinity stress tolerance in *Arabidopsis thaliana*	^36^
KSA- Red Sea coast	Endophytic bacteria *T. terrestris* and *Z. simplex, P. turgidum*	*Actinobacteria, Proteobacteria, Bacillus sp*	Increase in plant shoot and root weight under salt stress conditions	^37^
KSA- Al Jouf	Rhizosphere of *P. turgidum*	*Actinobacteria sp.*	Production of flavonoids, phytohormones, and siderophores, and reduction of detrimental effects of drought stress	^103^
KSA	Nodules of *Indigofera argentea*	*Enterobacter sp.*	Enhance the yield of alfalfa crops using saline irrigation in the field, and growth of the model plant * A. thaliana* in-vitro	^96^
KSA-Hada Al Sham	Rhizobacteria from the various desert plants including *Setaria viridis, Cenchrus ciliaris, P. antidotale, Amaranthus viridis,* and *Dichanthium annulatum*)	*Bacillus, Actinobacter,* and *Enterobacter*	Production of IAA, ACC deaminase, siderophores, and phosphatase. Enhancing the growth, nutrient uptake, and yield of alfalfa	^40^
KSA	Endophytic root nodules of *Indigofera argentea*	*Enterobacter sp.*	Production of Indole and siderophore as well as enhancing the growth and survival of * A. thaliana* under high salt and low water content	^39^
KSA-Hada Al Sham	The rhizosphere of wheat plants	*Azospirillum brasilense*	Nitrogen fixation and IAA production	^104^
KSA- Almadinah Almunawarah	Rhizobacteria from the various desert plants including *Zygophyllum simplex L., Peganum harmala L., Notoceras bicorne,* and *Cassia italica*	*Bacillus, Enterobacter* and *Pseudomonas*	Nitrogen fixation, production of ammonia, IAA, siderophores, solubilization of phosphate and zinc, and antagonistic potential against *F. oxysporum,* and *S. sclerotiorum*	^41^
KSA- Makkah	Rhizosphere and rhizoplane samples of different desert plants	*P. putida, P. fluorescens, B. cereus, Microccoucs sp.,* and *B. subtilis*	IAA production, increasing seed germination, plant height, shoot dry weight, and root elongation of Maize under laboratory conditions	^105^
KSA	Endophytes of *Plectranthus tenuiflorus*	*Bacillus spp., B. licheniformis, Paenibacillus spp., Pseudomonas spp.,* and *Acinetobacter calcoaceticus*	Extracellular enzymatic activity including protease, and cellulase production. Antimicrobial activities against *Staphylococcus aureus* and *Escherichia coli*	^106^
KSA-North Jeddah	Rhizosphere of pioneer plant species	*B. subtilis, B. amyloliquefaciens*, and *P. aeruginosa*	Antagonistic activity against the *P. aphanidermatum* seed protection against the phytopathogen, improved growth and biomass of cucumber	^75^

Hydrolytic enzymes are also considered an important mechanism by which bacteria fight phytopathogens^86,87^. Tests showed that most isolates were capable of protease production, and only (SALIR5) (*B. subtilis*) showed weak protease activity. Several microorganisms that produce extracellular proteases have shown promising effects against phytopathogens like *B. cinerea* ^88^. The cellulase activity test also showed that all of the selected isolates except SALIR5 were capable of degrading cellulose when it was provided as the sole source of carbon; this was indicated by the formation of a clear zone around the colonies. The reduction of radial growth in the in-vitro studies against the two phytopathogens could be linked to the production of hydrolytic enzymes that could lead to the degradation of fungal cell walls, and thus the formation of inhibition zones in dual culture plates. This is similar to a study where *B. subtilis* was used against many phytopathogens, and similar effects were noticed^89^.

Salinity stress is a common issue, especially in arid lands such as Qatar. Several studies showed that PGPR are capable of increasing plants’ tolerance to salt^90–92^. In the current study, all bacterial isolates were capable of growing in 5% NaCl salinity, and only one strain was found to grow weakly (SALIR5). Thus, further tolerance tests were carried out using 10% NaCl, and the growth was reduced compared to the 5%, yet most of the bacterial isolates were capable of growing and only two stains had weak growth (SALIR5) and (SOVERR2). According to the literature, numerous bacteria including *B. subtilis* could promote plant growth under both normal and salt-stress conditions^93,94^. Furthermore, when salinity stress is increased, plants increase the synthesis of ethylene to decrease their growth, this is done by the production of 1-aminocyclopropane-1-carboxylic acid (ACC). As mentioned previously, bacteria can metabolize ACC through ACC-deaminase, thus, promoting plant growth and reducing stress at the same time^95^. Several studies conducted across the Arabian Gulf have also identified several other PGPR strains with remarkable salt tolerance capabilities (Table 5). In Jizan, *C. muytjensii* exhibited increased salt tolerance in *Arabidopsis thaliana* and produced compounds inhibiting the growth of the phytopathogenic oomycete *Phytophthora infestans*^62^. Similarly, *Paenibacillus* sp. JZ16, isolated from *Zygophyllum simplex*, demonstrated the ability to enhance salinity stress tolerance in *A. thaliana*^36^. Along the Red Sea coast, endophytic bacteria *T. terrestris, Z. simplex*, and *P. turgidum* displayed increased shoot and root weight under salt stress conditions^37^. *Enterobacter* sp. SA187, isolated from nodules of *Indigofera argentea*, not only enhanced alfalfa crop yield under saline irrigation but also promoted the growth of *A. thaliana *in-vitro^96^. These studies show the significance of these PGPR strains in enhancing plant growth under saline conditions, offering valuable tools for agricultural practices in arid environments.

All the selected isolates were capable of ACC deaminase production except SALIR5. Another important trait of PGPR is the reduction of stress by decreasing ethylene levels. To achieve that, bacteria produce ACC deaminase that hydrolyses 1-aminocyclopropane-1-carboxylic acid (ACC) (which is a precursor of ethylene) into ammonia and α-ketobutyrate. Studies also suggest that bacteria with ACC activities could indirectly improve plant health by increasing the tolerances of the plants to various pressures including droughts, salinity, floods, and various phytopathogens^53,97^. In addition to that, ACC deaminase enzymes can help in the production of deep roots, leading to improved water acquisition in plants, especially in arid lands^98^.

The production of auxins including IAA is another direct mechanism employed by PGPR to enhance the growth of plants by increasing the surface area of roots through the growth of secondary roots^55,99^. Most rhizobacteria can synthesize IAA^100^, in the current study, 3 of the isolates tested positive for the production of IAA. These species increased the biomass of tomato seedlings in the presence and absence of phytopathogens as mentioned earlier.

In terms of ammonia production, it was found that all rhizobacteria tested positive as indicated by the change of color into deeper yellow/brown. LEMR3 (*B. subtilis*) and SAlSOR2 (*Bacillus* sp.) showed the highest level of ammonia production, and this could be linked to the improvement of growth noticed in the in-vivo studies mentioned earlier. Ammonia production is considered one of the essential traits due to its linkage to direct improvement of plant’s growth, this is because the produced ammonia could serve as a nitrogen supply to the host plant, and thus lead to biomass increase and shoot and root elongation^46,101^. In addition to that, studies showed that *B. subtilis* could be used effectively to control agricultural NH_3_ emissions, by reducing its volatilization, which is an important contribution to sustainable agriculture^102^.

It could be summarized from these characterization studies that locally isolated rhizobacteria exhibited multiple plant growth promoting and biocontrol traits. These bacteria produced hydrogen cyanide, enhanced phosphate solubilization, and secreted hydrolytic enzymes, showcasing their ability to increase nutrient availability and combat phytopathogens. The isolates displayed resilience in high salinity conditions and produced ACC deaminase. Furthermore, their production of auxins like indole-3-acetic acid (IAA) and ammonia demonstrated their role in promoting plant growth as was shown previously in greenhouse studies. The limited research on PGPR in the Arabian Gulf region, especially in Qatar, is strikingly apparent as indicated in (Table 5). Furthermore, the existing studies predominantly focus on specific in-vitro experiments, with only a few studies exploring in-vivo exploration of PGPR in agricultural contexts. This research, therefore, fills a significant gap in the existing knowledge. Serving as a pioneering study, it establishes a foundational understanding of PGPR's role in agriculture within Qatar and neighboring countries. By showcasing the diverse capabilities of locally isolated rhizobacteria, this study not only contributes valuable data to the global scientific community but also lays the groundwork for future agricultural advancements in the region.

## Conclusions

The current study showed that PGPR belonging to the genus *Bacillus* collected from the rhizosphere of Qatari plants possesses anti-fungal and plant growth-promoting features. These findings were substantiated through a series of in-vitro and in-vivo experiments involving two plant pathogens and a host plant, namely, tomato. The bioassays explicitly demonstrated that these bacteria possess inherent qualities that not only combat phytopathogens but also enhance plant growth, both in the presence and absence of these pathogens. The significance of these indigenous species lies not only in their ability to promote plant growth but also in their resilience in the harsh local environment, marked by high salinity and elevated temperatures. Given the diverse characteristics observed even within the same species, it becomes imperative to intensify the study and isolation of more rhizobacteria. It is also essential for future studies to include larger numbers of strains and wider groups of microorganisms especially in in-vivo studies under field conditions to establish their efficiency under varying environmental conditions. This exploration is essential to identify strains with exceptional plant growth-promoting and stress tolerance traits, making them promising bioinoculants for sustainable agriculture practices in arid lands.

## Data Availability

The datasets generated during and/or analyzed during the current study are available from the corresponding author on reasonable request.
